# Interpretable Machine Learning Model for Survival Prediction in Pediatric Adrenocortical Tumors

**DOI:** 10.1210/jendso/bvaf177

**Published:** 2025-11-18

**Authors:** Antje Redlich, Elisabeth Pfaehler, Marina Kunstreich, Maximilian Schmutz, Christoph Slavetinsky, Eva Jüttner, Paul-Martin Holterhus, Gert Warncke, Christian Vokuhl, Jörg Fuchs, Stefan A Wudy, Michaela Kuhlen

**Affiliations:** Department of Pediatrics, Pediatric Hematology/Oncology, Otto-von-Guericke-University, Magdeburg D-39120, Germany; Institute for Neuroscience and Medicine 4, INM-4, Forschungszentrum Jülich GmbH, Jülich D-52428, Germany; Department of Pediatrics, Pediatric Hematology/Oncology, Otto-von-Guericke-University, Magdeburg D-39120, Germany; Pediatrics and Adolescent Medicine, Faculty of Medicine, University of Augsburg, Augsburg D-86156, Germany; Hematology and Oncology, Faculty of Medicine, University of Augsburg, Augsburg D-86156, Germany; Bavarian Cancer Research Centre (BZKF), Augsburg D-86156, Germany; Department of Pediatric Surgery and Pediatric Urology, University Children's Hospital, Eberhard-Karls University, Tuebingen D-72076, Germany; Department of Pathology, University Hospital Schleswig-Holstein, Kiel D-24105, Germany; Department of Pediatrics, Pediatric Endocrinology and Diabetes, University Hospital of Schleswig Holstein (UKSH), Kiel D-24105, Germany; Department of Pediatrics, Pediatric Intensive Care Unit, Otto-von-Guericke-University, Magdeburg D-39120, Germany; Section of Pediatric Pathology, Department of Pathology, University Hospital Bonn, Bonn D-53127, Germany; Department of Pediatric Surgery and Pediatric Urology, University Children's Hospital, Eberhard-Karls University, Tuebingen D-72076, Germany; Steroid Research & Mass Spectrometry Unit, Laboratory for Translational Analytics, Pediatric Endocrinology & Diabetology, Center of Child and Adolescent Medicine, Justus Liebig University Giessen, Giessen D-35392, Germany; Pediatrics and Adolescent Medicine, Faculty of Medicine, University of Augsburg, Augsburg D-86156, Germany; Bavarian Cancer Research Centre (BZKF), Augsburg D-86156, Germany

**Keywords:** children and adolescents, adrenocortical tumors, interpretable machine learning model, survival prediction

## Abstract

**Purpose:**

Pediatric adrenocortical tumors (pACTs) are rare and clinically heterogeneous. Existing risk stratification systems rely on fixed thresholds and linear assumptions, which may limit their prognostic accuracy—particularly for nonmetastatic, locally advanced cases. We aimed to develop an interpretable machine learning (ML) model for individualized survival prediction using only routine clinical features.

**Methods:**

We retrospectively analyzed 97 patients with pACT from the German Pediatric Oncology Hematology-Malignant Endocrine Tumors Registry (1997-2024). An Extreme Gradient Boosting Cox proportional hazards model was trained using 4 features—tumor volume, distant metastases, pathologic T stage, and resection status—identified via systematic feature evaluation across 11 737 model combinations. Performance was assessed using a stratified 80/20 train–test split, 500 bootstrap iterations, and Harrell's concordance index (C-index). SHapley Additive exPlanations (SHAP) were used for interpretability.

**Results:**

The model achieved strong prognostic performance (test-set C-index: 0.925; bootstrap mean: 0.891, 95% confidence interval: 0.817-0.946). SHAP analysis confirmed the dominant influence of metastatic status, followed by tumor volume, T stage, and resection status. The model uncovered nonlinear and additive effects, including a SHAP- and bootstrap-guided tumor volume cut-off (190 mL, 95% confidence interval 127-910 mL) that only slightly differed from conventional thresholds. Stratification remained robust in subgroups, including nonmetastatic patients with advanced local disease.

**Conclusion:**

This interpretable ML model enables individualized survival prediction in pACT using only routine clinical data. It offers a clinically accessible and clinically meaningful complement to existing scoring systems, particularly in patients with ambiguous risk profiles who may benefit from more personalized management.

Pediatric adrenocortical tumors (pACTs) are exceptionally rare endocrine neoplasms, with an estimated incidence of 0.2 to 0.3 per million children and adolescents per year, rising to over 3 per million in endemic regions such as southern Brazil due to the founder variant p.R337H in *TP53* [1, 2]. Clinical presentation and prognosis vary widely, ranging from small, hormonally active tumors with indolent behavior to large or metastatic carcinomas with aggressive clinical course. Complete surgical resection offers favorable outcomes in small, localized tumors; however, recurrence occurs in up to 50% of patients with large or incompletely resected lesions [3, 4]. Outcomes for patients with metastatic or relapsed disease remain dismal, with overall survival below 20% [5-9].

Current treatment protocols typically escalate intensity only in patients with locally advanced or residual disease or overt metastases [5, 10]. However, a subset of patients with initially localized but biologically aggressive tumors may be undertreated based on existing criteria [3, 5]. The prognostic stratification of these pACTs remains particularly challenging.

To address this, several clinical, histopathological, and combined prognostic scoring systems have been proposed for pACTs. These include the Wieneke score [11], the Children's Oncology Group (COG) staging system [5], the 5-item microscopic score [12], the Reticulin algorithm [13], and the pediatric S-GRAS (pS-GRAS) score [14]. Despite these efforts, all current tools are fundamentally rule-based and may not capture the complex, nonlinear interactions among clinical, pathological, and biological factors—especially in rare, heterogeneous diseases like pACT.

In contrast, machine learning (ML) approaches have begun to reshape prognostic modeling in adult adrenocortical carcinoma (ACC). Most published studies to date have incorporated molecular data (eg, transcriptomics, DNA methylation, proteomics) alongside clinical variables to classify tumor subtypes and predict outcomes [15-19]. Only one study has developed an ML-based prognostic tool exclusively from routinely available clinical patient data, using the S-GRAS score components to estimate individual survival probabilities in adults [20]. Another ML-based model was developed using data from the SEER (Surveillance, Epidemiology, and End Results) Registry, which also includes only clinical variables but with more limited clinical granularity, reflecting its origin as a population-level epidemiological dataset [21]. To our knowledge, no ML-based prognostic models currently exist for pACTs. Given the ultra-rare nature of the disease, the aforementioned clinical challenges, and the global disparities in access to molecular testing, this is a critical gap [22]. ML models based solely on universally collected clinical parameters have the potential to provide globally applicable, accessible, and individualized prognostic insights.

Among the various ML algorithms suited for clinical prediction, Extreme Gradient Boosting (XGBoost) has demonstrated strong performance and reliability in structured medical datasets [23-25]. It builds ensembles of decision trees with integrated regularization, offering robustness and flexibility in modeling complex feature interactions while preventing overfitting. In this study, we developed the first interpretable ML-based survival model for pACTs using only routinely collected clinical variables. To enhance model transparency and interpretability, we used SHapley Additive exPlanations (SHAP) to visualize the relative contribution of each feature to the model's output [26].

## Methods

### Study Population and Data Source

This retrospective study included pediatric patients (<18 years) diagnosed with adrenocortical tumors (ACTs) enrolled in the German Pediatric Oncology Hematology–Malignant Endocrine Tumors Registry between 1997 and December 2024. Inclusion criteria required documentation of diagnosis (ACC or adrenocortical tumor of undetermined malignant potential), age at diagnosis, and outcome status (survival or death due to disease progression). After applying these criteria, 115 patients were eligible, of whom 97 had complete data for the selected model variables and were included in the final analysis.

### Ethical Approval and Patient Consent

This study was conducted in accordance with the Declaration of Helsinki and approved by the ethics committees of the University of Luebeck (IRB 97125) and Otto-von-Guericke-University Magdeburg (IRBs 174/12 and 52/22), Germany. Written informed consent was obtained from all patients and/or legal guardians at the time of registry enrollment, in accordance with national regulations. All data were pseudonymized prior to analysis.

### Outcome Definition

The primary endpoint was disease-specific mortality, defined as death attributable to ACT progression. Survival time was calculated from the date of diagnosis to death or last follow-up. Patients alive at last follow-up were right-censored.

### Variable Selection

Fifteen clinical and pathological variables were initially considered based on established or suspected prognostic relevance in pACTs. These included age at diagnosis (years, continuous), age group (<10 years vs ≥10 years, binary), sex (binary), interval from symptom onset to diagnosis (weeks, continuous), virilization (binary), Cushing syndrome (binary), tumor size (cm, continuous), tumor volume (ml, continuous), pathological tumor (pT) stage (categorical: T1-T4), nodal status (binary), clinical metastasis status (cM; binary), Ki-67 index (percent, continuous), tumor necrosis (binary), vascular invasion (binary), and resection status after initial surgery (R0 vs other, binary).

Clarification of nodal and metastatic status: when both clinical and pathological nodal information were available, the pathological nodal superseded the clinical nodal; if pathological nodal assessment was unavailable, clinical nodal (imaging) was used. cM was determined clinically by imaging per registry standards at diagnosis.

### Model Development and Feature Selection

To identify the most suitable prognostic model, we systematically evaluated all valid combinations of 3 to 7 features drawn from the 15 a priori candidates, excluding redundant pairings (eg, tumor size with volume, continuous age with age group). This yielded 11 737 candidate models (3 features: 429; 4 features: 1210; 5 features: 2442; 6 features: 3630; 7 features: 4026).

For each feature set, we performed a single stratified 80/20 train–test split (preserving the event/censoring ratio) using a fixed random seed (random_state = 42) for reproducibility; the split was rerun per feature set to avoid coupling the evaluation across different sets. All model development (feature-set evaluation and hyperparameter tuning) was restricted to the training data.

Models were fitted with XGBoost using a Cox proportional hazards objective (objective=“survival:cox”, eval_metric=“cox-nloglik”). Survival labels were encoded as a signed time vector to interface with scikit-learn. A custom scorer was implemented to recover time and event indicators, and the concordance index (C-index) was computed using sksurv.metrics.concordance_index_censored. function was used to compute the C-index. Within the training set, RandomizedSearchCV with 3-fold cross-validation was applied to optimize hyperparameters by maximizing the cross-validated C-index. The best hyperparameters for each feature set were then fixed and used for subsequent evaluations.

### Model Ranking and Final Feature Selection

For ranking, each candidate model's performance was summarized by the mean C-index from an internal bootstrap resampling of the training set (see Internal Validation for details). In case of ties or negligible differences [overlapping 95% confidence intervals (CIs) and <0.01 absolute difference in mean C-index], we preferred the more parsimonious feature set and, secondarily, the single-run test-set C-index.

The final model retained 4 features based on predictive performance and clinical interpretability:

- Pathologic T stage
- Presence of distant metastases
- Resection status after initial surgery (R0 vs other)
- Tumor volume (mL)

Variables such as continuous age, binary age group (<10 vs ≥10 years), Ki67, and hormone secretion were evaluated during model selection but were not retained because they did not add measurable prognostic value once the aforementioned 4 features were included.

Following final feature selection, we adopted a complete-case approach for these 4 variables. Of the 115 eligible patients, 97 had nonmissing values for tumor volume, resection status, pT stage, and metastases status and were included in model training and evaluation. Given that these variables reflect core disease biology and surgical outcome, we did not impute them, as extrapolation was deemed neither reliable nor appropriate.

### Hyperparameter Optimization

The optimal hyperparameter combination, selected via 3-fold cross-validation on the training set and held fixed for all subsequent model retraining, was

- learning_rate: 0.03
- max_depth: 4
- n_estimators: 150
- min_child_weight: 1
- subsample: 0.8
- colsample_bynode: 0.5
- reg_alpha: 0.01
- reg_lambda: 0.1

This parameter set was applied consistently during bootstrap retraining of the final model.

### Internal Validation and Model Evaluation

Because of the limited sample size, we avoided relying on a single train–test split for model evaluation. Instead, we applied a nested and bootstrapped validation strategy to assess generalizability and correct for potential optimism in performance estimates. For each candidate feature set, hyperparameters were tuned using 3-fold cross-validation within the training set only (RandomizedSearchCV), ensuring that model optimization remained fully independent of the test set.

The final model was then evaluated on a fixed, untouched test set (20% of the original cohort), which had been held out from the beginning of model selection. To obtain optimism-corrected performance estimates, we conducted 500 bootstrap iterations. In each iteration, 80% of the training data was resampled with replacement, and the model was retrained using the fixed hyperparameters. Performance was evaluated on the independent test set, and the mean Harrell's C-indices with 95% CIs were computed across all iterations.

To further probe internal robustness, we also ran nested cross-validation across the entire dataset, with outer folds used for evaluation and inner folds for hyperparameter tuning; the distribution of resulting C-indices is shown in Fig. S1 [27]. Finally, to examine whether the available cohort size was sufficient for stable training, we constructed a learning curve by incrementally increasing the proportion of training data and recording model performance (Fig. S2 [27]).

Stratified splitting preserved event distribution, resulting in 77 patients (23 events) in the training set and 20 patients (6 events) in the independent test set. This combined nested, bootstrapped, and learning-curve evaluation provided a robust internal estimate of model discrimination while mitigating the risk of overfitting.

### Brier Score and Calibration Analysis

To complement discrimination metrics with a measure of calibration, we computed the time-dependent Brier score on the independent test set. The Brier score reflects the squared difference between predicted survival probability and observed survival status at a given time point, thereby integrating aspects of both discrimination and calibration into a single metric: lower values indicate better performance.

To obtain an aggregate measure of predictive error across the entire follow-up period, we calculated the integrated Brier score (IBS), which integrates the Brier score across all observed time points and provides a summary of the model's average prediction error over time. All computations were performed using the *pySurvival* and *sksurv* packages. No additional bootstrapping was applied to the IBS calculation.

### Model Explainability Using SHAP

To enhance clinical interpretability and transparency of the survival model, we applied SHAP, a cooperative game theory-based approach that quantifies the contribution of each input feature to an individual prediction. For each patient, SHAP values indicate whether a specific feature increases or decreases their predicted risk and by what magnitude.

Two types of visualizations were generated: SHAP summary plots, which display the direction and magnitude of feature effects across the entire cohort, and SHAP dependence plots, which illustrate how specific feature values (eg, tumor volume) influence model output and help identify potential nonlinear relationships or threshold effects. These explainability tools provide both global insights into feature relevance and individualized interpretation of risk profiles, thereby supporting clinical applicability and decision-making.

### Determining and Evaluating a Tumor Volume Cut-off

Given the clinical relevance of tumor size, we sought to identify an interpretable, data-driven threshold in tumor volume that best discriminated between high- and low-risk patients. SHAP values for tumor volume were first extracted from the fitted XGBoost–Cox model, representing the marginal contribution of tumor volume to predicted risk for each patient. We then systematically scanned the distribution of tumor volumes to identify the threshold that maximally separated patients into high- and low-risk groups.

To evaluate the stability of this threshold, we conducted 500 bootstrap resampling iterations with model refitting and calculated a 95% CI for the optimal cut-off. In addition, repeated stratified cross-validation (5 × 5) was used to assess consistency across different training folds. To further characterize interactions, a SHAP dependence analysis examined the joint influence of tumor volume and metastatic status.

Finally, the discriminatory strength of the cut-off was tested by dichotomizing the full cohort at the derived threshold and computing the C-index in both training and test sets, thereby providing an independent check of its prognostic utility.

The stability of the SHAP-informed, bootstrap-derived tumor volume cut-off was further assessed by visualizing its distribution across 500 bootstrap iterations, the corresponding empirical cumulative distribution, and repeated cross-validation resamples (Figs. S3-S5 [27]).

### Computation of Absolute Survival Probabilities

Although the XGBoost model was trained to estimate relative risks (log-hazards), we also derived absolute survival probabilities $\hat{S}(t\;x)\;$ for each patient to enhance clinical interpretability. For this purpose, we applied the Breslow estimator, which permits survival curve estimation even in complex, nonlinear models.

For each patient *i*, the model's output $f(x_{i})\;$ was transformed into a risk score:


$$r_{i}=\exp\;(\;f(x_{i}))$$

At each observed event time $t_{\left(k\right)}$, the cumulative baseline hazard was estimated as:


$$\hat{H_{0}}(t_{\left(k\right)})=\underset{l:t_{\left(l\right)}\leq t_{\left(k\right)}}{\sum }\frac{d_{l}}{\sum_{\;j\in R_{l}}\;r_{j}}$$

where $d_{l}$ is the number of events at time $t_{\left(l\right)}$ and $R_{l}$ is the set of patients still at risk at that time.

The baseline survival function was then computed as:


$$\hat{S_{0}}(t)=\exp\;[-\hat{H_{0}}(t)]$$

Finally, the individual survival probability for patient *i* was given by:


$$\hat{S}(t|x_{i})=\hat{S_{0}}{\left(t\right)}^{\;r_{i}}$$

This approach enables the generation of personalized survival curves, offering a more intuitive clinical presentation of risk beyond relative hazard estimates.

### Subgroup Analysis by COG Stage

To explore the clinical utility of the ML model within established staging frameworks, we performed subgroup analyses according to COG stage, the most widely used system for pACTs.

Within each stage, patients were stratified into ML-high and ML-low risk groups based on the stage-specific median of the predicted risk scores. Kaplan–Meier survival curves were generated for these subgroups and compared using the log-rank test. When survival differences reached statistical significance, hazard ratios (HRs) with 95% CIs were reported.

Because sample sizes within individual stages were small, these analyses were performed on the entire cohort (training and test sets combined) and should be considered exploratory. Nonetheless, they provide insight into whether the ML-derived risk score adds prognostic resolution beyond traditional staging.

## Results

### Patient Characteristics

Of the 115 eligible pediatric patients with ACTs, 97 patients with complete data for the selected model features were included in the final analysis. The cohort comprised 63 females (64.9%) and 34 males (35.1%); 85 patients (87.6%) were diagnosed with ACC, and 12 (12.4%) with adrenocortical tumor of undetermined malignant potential. The median age at diagnosis was 5.1 years (range: 0.2-17.8 years). A detailed summary of clinical and pathological characteristics is presented in Table 1.

**Table 1.** Baseline clinical and pathological characteristics of the pediatric adrenocortical tumor cohort (n = 97)

Parameter	Metrics
Age at diagnosis, years	
Median	5.1
Range	0.2-17.8
Sex, n (%)	
Female	63 (64.9)
Male	34 (35.1)
Diagnoses, n (%)	
ACC	85 (87.6)
ACx	12 (12.4)
Staging, n (%)	
pN1	9/97 (9.3)
cM1	17/97 (17.5)
COG	1: 18 (21.4) 2: 17 (20.2) 3: 32 (38.1) 4: 17 (20.2)
Tumor size, cm	
Median	8.8
Range	1.0-30.0
Ki67 index, %	
Median	20.0
Range	1.0-80.0
Vascular invasion, n (%)	
65/84 (77.4)	
Follow-up duration (all patients), years	
Median	3.7
Range	0-20.7
Outcome, n (%)	
Alive	68 (70.1)
Deceased	29 (29.9)

Abbreviations: ACC, adrenocortical carcinoma; ACx, adrenocortical tumor of undetermined malignant potential; cM, clinical metastatic status; COG, Children's Oncology Group; pN, pathological nodal status.

At the time of analysis, 29 patients (29.9%) had died due to disease progression. The median follow-up for surviving patients was 7.0 years (range, 0-20.7).

### Model Performance

The final XGBoost survival model was trained using 4 clinical features: pT stage, presence of distant metastasis, resection status after first surgery, and tumor volume. These variables were identified from 11 737 evaluated feature combinations, ranging from 3 to 7 variables, using a systematic model-selection process prioritizing predictive accuracy and parsimony.

**Figure 1.**
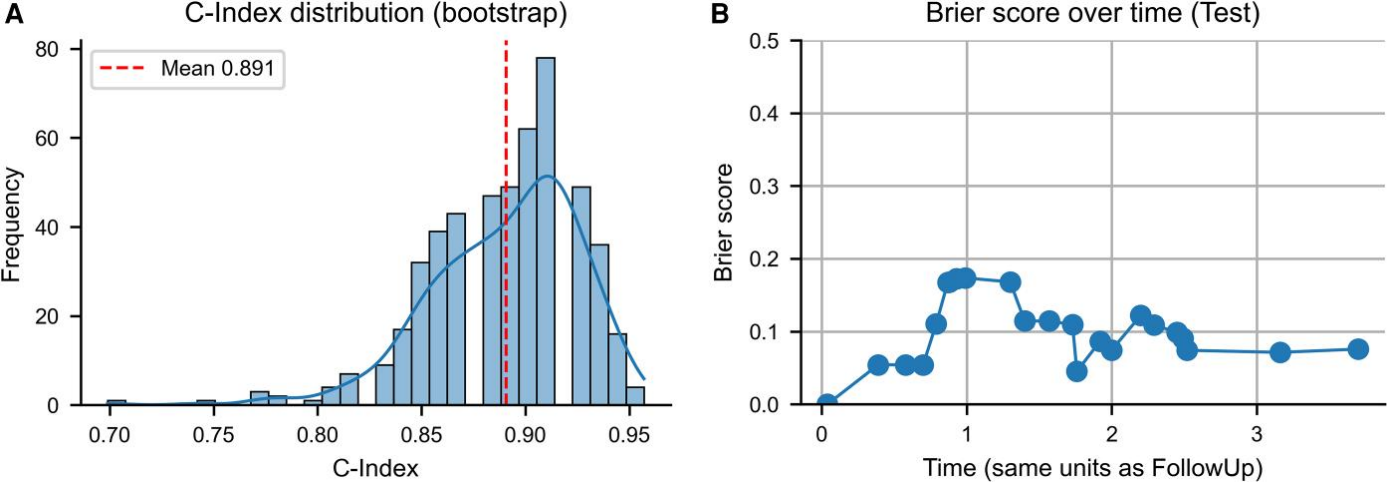
Model performance of the final XGBoost survival model. (A) Histogram of C-index values across 500 bootstrap iterations. Each value represents model performance on the fixed, independent test set after retraining on a resampled training set. The dashed line marks the mean C-index of 0.891. The model achieved a C-index of 0.925 on the original test set, demonstrating excellent discriminative ability. (B) Time-dependent Brier scores on the independent test set. The Brier score quantifies the prediction error at different time points, with lower values indicating better accuracy. The integrated Brier score was 0.09, reflecting strong overall calibration and predictive performance over time. Abbreviation: C-index, concordance index; XGBoost, Extreme Gradient Boosting.

On the independent test set, the model achieved a C-index of 0.925, indicating excellent discriminative ability. Across 500 bootstrap runs, the mean C-index was 0.891, with a 95% CI of 0.817 to 0.946 (Fig. 1A).

To further evaluate overall predictive accuracy, we calculated the time-dependent Brier score on the test set. The prediction error remained consistently low throughout the follow-up period (Fig. 1B). The IBS was 0.09, reflecting very good calibration and average prediction error over time.

Nested cross-validation confirmed the robustness of the model. Across 10 outer folds (with inner 3-fold tuning), the mean C-index was 0.78 (range 0.70-0.90) (Fig. S1 [27]). A learning curve showed performance stabilizing once ∼60% of the training data was used (Fig. S2 [27]), supporting that the available cohort size is sufficient for model convergence.

### Model Interpretation via SHAP

To enhance interpretability and clinical transparency, we applied SHAP to quantify feature contributions to the predicted log-risk.

**Figure 2.**
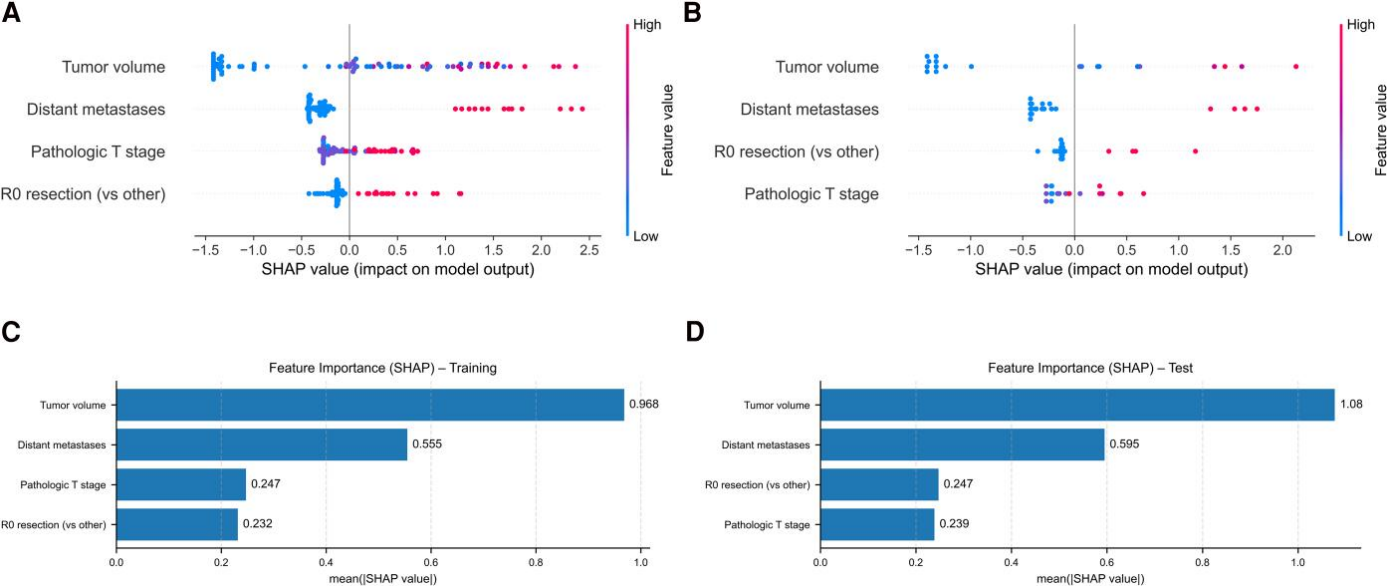
SHAP-based model interpretation in training and test datasets. (A, B) SHAP summary plots showing the directional impact of each input feature on the predicted mortality risk in the training (A) and independent test (B) datasets. Each dot represents a single patient. Color encodes the feature value (red = high, blue = low); position along the x-axis reflects the SHAP value (positive = increased risk). (C, D) Bar plots of mean absolute SHAP values in the training (C) and test (D) datasets, ranking features by their average contribution to model predictions. Tumor volume and distant metastases showed the strongest and most consistent influence, followed by resection status and pathological tumor stage. Abbreviation: SHAP, SHapley Additive exPlanations.

In the SHAP summary plot for the training dataset (Fig. 2A), tumor volume displayed a broadly monotonic association with risk; distant metastases showed a uniformly strong positive impact; pT stage contributed in an ordinal fashion; and resection status (R0 vs R1/R2) had a clear categorical effect, with incomplete increasing risk (with greater variability than metastases). These patterns were reproduced in the test set (Fig. 2B). Feature-importance rankings by mean absolute SHAP values (Fig. 2C and 2D) consistently placed tumor volume and metastatic status as the dominant predictors, followed by resection status and pT stage. The stability of global feature effects across 500 bootstraps is shown in Fig. S6 [27] (mean ± SD SHAP values), underscoring robust importance ordering.

### Generalizability and Robustness in Independent Test Data

Replicating SHAP analysis in the independent test set yielded concordant effect directions and relative magnitudes across all 4 features (Figs. 2B and 2D), supporting the stability and transferability of learned patterns despite smaller sample size.

### Feature-specific SHAP Dependence Analysis

**Figure 3.**
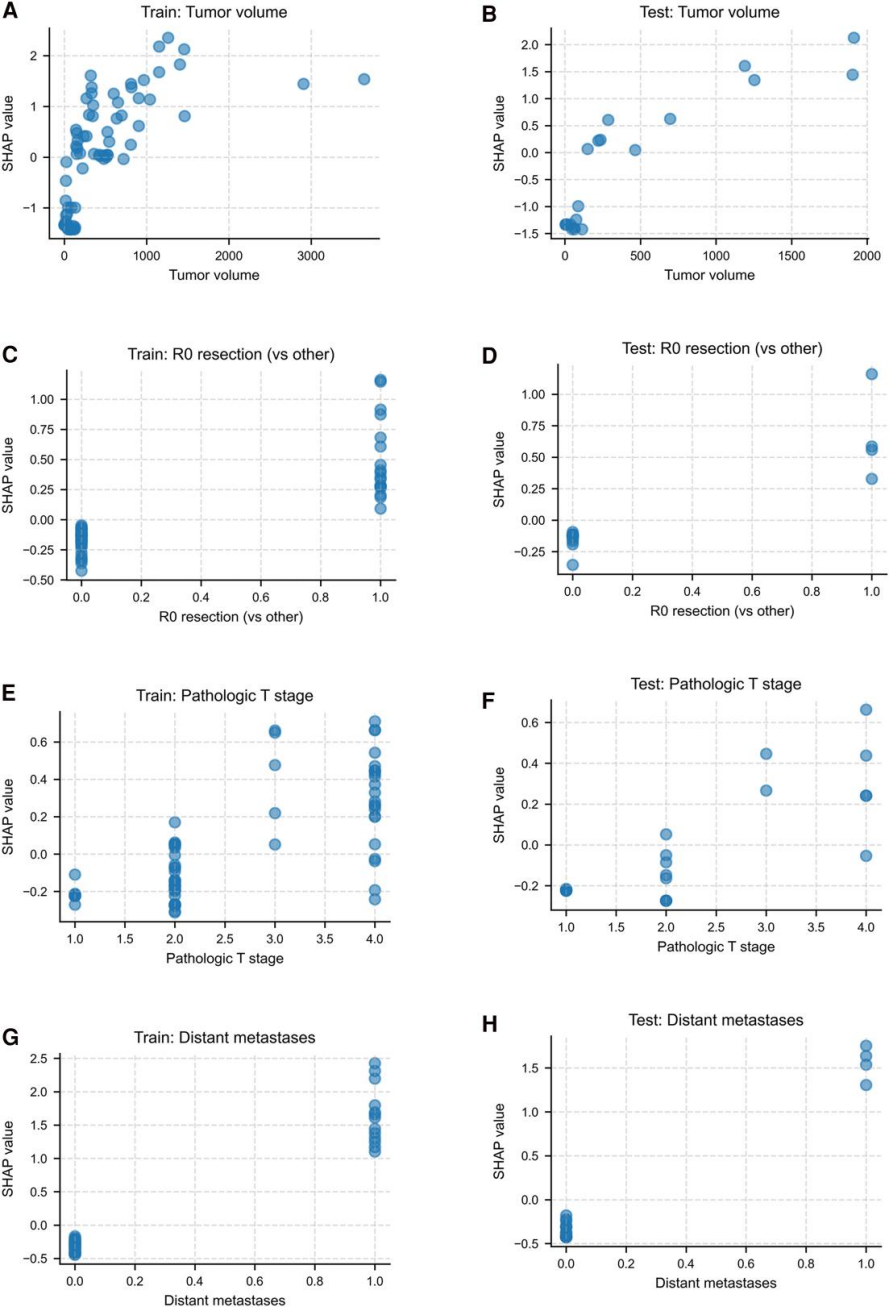
SHAP dependence plots for all model features in training and test datasets. Each panel pair displays the relationship between actual feature values and their corresponding SHAP values (predicted risk contribution). Left column: training data; right column: test data. (A, B) Tumor volume exhibits a steep risk increase up to ∼1000 mL, plateauing at higher volumes. (C, D) Resection status shows a clear binary risk split, with incomplete resections associated with higher risk. (E, F) Pathologic T stage demonstrates a progressive risk increase with advancing tumor stage. (G, H) Distant metastases yield the highest SHAP contributions, clearly distinguishing cM1 from cM0 cases. These plots highlight robust, clinically intuitive relationships between model features and predicted mortality risk. Abbreviations: cM, clinical metastatic status; SHAP, SHapley Additive exPlanations.

Feature-specific SHAP dependence plots provided detailed insights into how individual predictors influenced mortality risk (Fig. 3). Tumor volume showed a nonlinear effect, with SHAP values rising steeply up to approximately 1000 mL and then plateauing, indicating diminishing incremental risk at very large volumes. Resection status displayed a clear binary pattern, with incomplete resection associated with substantially increased mortality risk. pT stage demonstrated a graded, monotonic increase in SHAP values with advancing local extension, consistent with an ordinal, stepwise contribution to risk; however, its overall effect size remained smaller than that of tumor volume and metastatic status. Distant metastasis at diagnosis exerted a strong positive SHAP effect, highlighting its dominant prognostic contribution.

To further examine potential interactions, SHAP dependence plots for tumor volume were stratified by metastatic status. These showed parallel, monotonic increases in risk, indicating that the prognostic effect of tumor volume was not modified by metastatic status (Fig. S7 [27]).

Across 500 bootstrap iterations, tumor volume and metastatic status consistently emerged as the dominant predictors of mortality risk, with substantially higher mean SHAP values compared to pT stage and resection status (Fig. S6 [27]). This confirms the stability of feature importance rankings despite the limited cohort size.

Calibration was additionally evaluated through visual plots at 1-year and 3-year survival, generated from the independent test set. These plots showed reasonable agreement between predicted and observed survival probabilities, despite the small sample size (n = 20; 6 events) and are presented in Figs. S8 and S9 [27].

### Tumor Volume Cut-off Identification and Prognostic Evaluation

**Figure 4.**
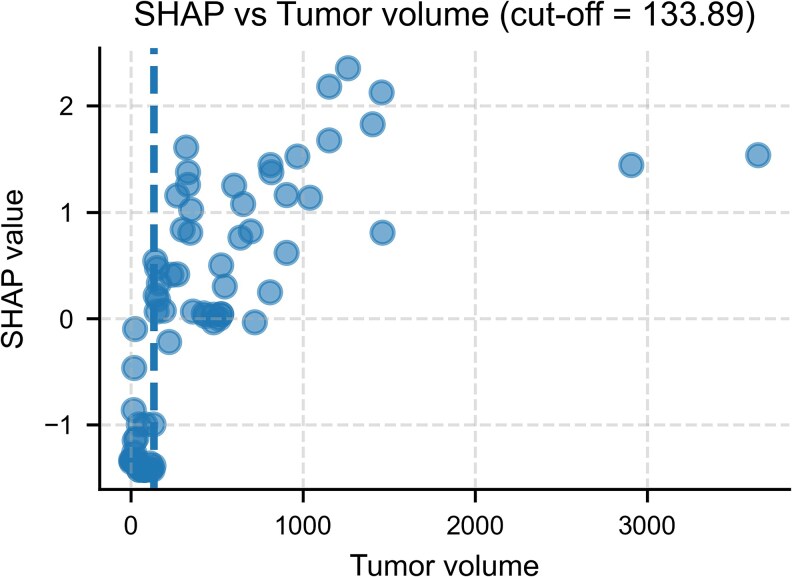
SHAP-guided identification of a tumor volume threshold. Scatter plot of SHAP values vs tumor volume (mL) in the training dataset. Each dot represents 1 patient. The vertical dashed line marks the optimal cut-off at 133.9 mL, above which SHAP contributions to predicted mortality risk become predominantly positive. This threshold provides a clinically interpretable reference point derived from the model. Abbreviation: SHAP, SHapley Additive exPlanations.

To provide a clinically interpretable reference, we derived a SHAP-guided tumor volume threshold that marks the onset of consistently positive (risk-increasing) SHAP contributions. In the single training run, this cut-off was 133.9 mL (95% CI: 119.7-138.8 mL), separating patients with predominantly positive vs neutral/negative SHAP values (Fig. 4). When used to dichotomize patients, the cut-off yielded a C-index of 0.669 (training) and 0.790 (test), indicating meaningful prognostic separation.

Robustness checks showed a consistent low-volume signal: across 500 bootstrap refits, the median cut-off was ∼190 mL (95% CI: 127-910 mL), with >80% of cut-offs <400 mL (Figs. S3 and S4 [27]). Repeated 5 × 5 cross-validation similarly centered around ∼190 mL, with a narrow interquartile range and occasional higher estimates attributable to small-sample variability (Fig. S5 [27]).

### Subgroup Analysis by COG Stage

**Figure 5.**
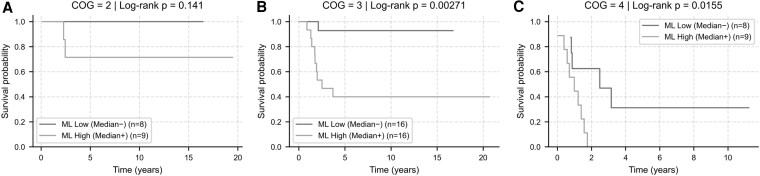
Risk stratification within COG stages using ML-derived risk scores. Kaplan–Meier survival curves for ML-high (bright) vs ML-low (dark) patients within each COG stage subgroup: (A) COG stage 2 (n = 17): No significant difference observed (log-rank *P* = .141). (B) COG stage 3 (n = 32): ML-high patients demonstrated significantly worse survival (log-rank *P* = .0027; HR = 12.0; 95% CI: 1.52-95.3). (C) COG stage 4 (n = 17): ML stratification identified 2 prognostically distinct groups (log-rank *P* = .0155; HR = 4.6; 95% CI: 1.20-17.5). These results highlight the potential of the ML model to refine prognosis within clinically relevant subgroups, particularly in intermediate- and high-risk COG stages. Abbreviation: CI, confidence interval; COG, Children's Oncology Group; HR, hazard ratio; ML, machine learning.

Within the COG staging framework, stage-specific median ML risk scores separated outcomes in advanced disease. In COG stage 3 (n = 32), ML-high patients demonstrated significantly worse survival than ML-low patients (log-rank *P* = .0027; HR = 12.0; 95% CI: 1.52-95.3). Similarly, in COG stage 4 (n = 17), the ML risk score distinguished two prognostically distinct groups (log-rank *P* = .0155; HR = 4.6; 95% CI: 1.20-17.5). No significant separation was observed within COG stages 1 or 2, consistent with very low event counts and overall favorable prognosis. Kaplan–Meier curves are shown in Fig. 5.

### Individualized Predictions in Representative Clinical Scenarios

**Figure 6.**
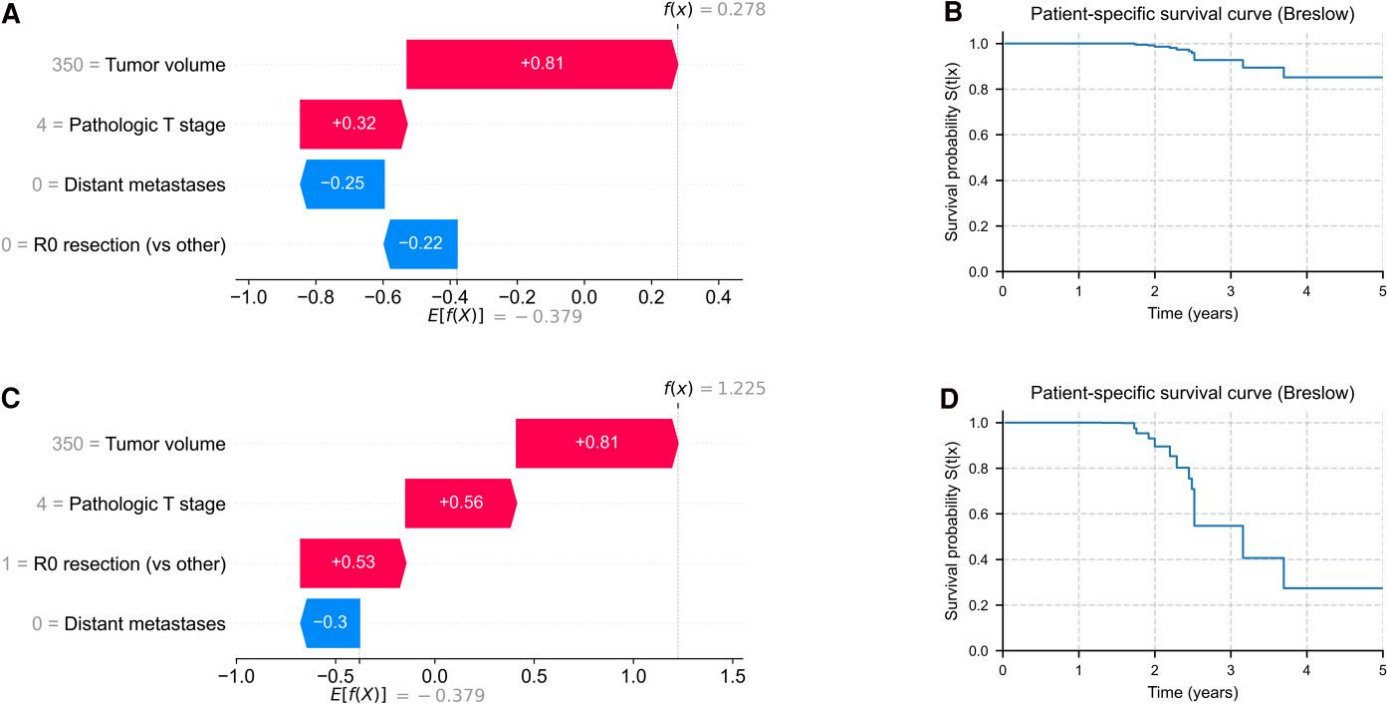
Individualized survival predictions using SHAP force plots and model-derived survival curves. (A, B) Patient 1: pT4 tumor, tumor volume 350 mL, cM0, R0 resection. SHAP plot (A) shows moderate risk contributions offset by protective features, resulting in a high predicted survival probability (B). (C, D) Patient 2: pT4 tumor, tumor volume 350 mL, cM0, R1/2 resection. SHAP plot (C) shows additive contributions of all adverse features, yielding a substantially elevated risk and poor predicted survival (D). These examples demonstrate the model's capacity for personalized, interpretable prognostication based on the full clinical context. Abbreviation: cM, clinical metastatic status; pT, pathological tumor; SHAP, SHapley Additive exPlanations.

Finally, two locally advanced, nonmetastatic ACC cases illustrate individualized predictions (Fig. 6). For patient 1 (Fig. 6A and 6B), this was pT4 tumor, cM0, R0, 350 mL. Tumor volume and T stage increased risk, whereas the absence of metastases and R0 resection reduced risk. Predicted log-risk [f(x) = 0.278; estimated 3-year survival ∼88%] (Fig. 6B). For patient 2 (Fig. 6C and 6D), this was pT4, cM0, R1/R2, 350 mL. Incomplete resection added substantial risk on top of high volume and T stage, yielding f(x) = 1.225 and an estimated 3-year survival ∼33%.

These scenarios demonstrate how the model incorporates nonlinear and additive effects across routine clinical features to produce transparent, patient-specific risk estimates.

## Discussion

In this study, we present the first interpretable ML survival model for pACTs trained exclusively on routinely available clinical features. Using a gradient-boosted Cox proportional hazards approach, the model achieved high predictive accuracy (C-index 0.925 on the independent test set; optimism-corrected mean 0.891, 95% CI: 0.817-0.946). These results demonstrate that individualized survival risk prediction in pACT is feasible even without molecular data, supporting broad applicability, including in resource-limited settings.

Model transparency was ensured through SHAP-based interpretation [26]. Tumor volume, distant metastases, pathologic T stage, and resection status emerged as the final predictive features, each reflecting known markers of tumor burden and operability [5, 10, 28]. Importantly, SHAP revealed nonlinear and interaction-based effects not captured by traditional categorical staging systems [5, 11-14]. Feature importance rankings were highly stable across 500 bootstrap iterations (Fig. S6 [27]), underlining the robustness of model interpretation.

Tumor volume showed a steep, nonlinear association with risk, plateauing above ∼1000 mL. A SHAP-guided, bootstrap-estimated cut-off identified ∼190 mL as the point where volume began to contribute consistently to risk, slightly lower than the conventional 200 mL threshold [6]. Subgroup-specific cut-offs (eg, by age or metastatic status) were not attempted due to underpowered sample sizes. Similarly, pathologic T stage demonstrated a graded increase in risk, resection status exerted a categorical effect, and metastasis at diagnosis was the single most influential predictor [6, 29].

Biological variables such as age, hormone secretion, and Ki-67 index, though clinically relevant and used in scores like pS-GRAS [28], were systematically tested but did not improve performance beyond the final 4 features. Particularly, age at diagnosis has long been recognized as a key prognostic factor in pACT and forms part of established risk frameworks. In our analyses, both continuous and categorical representations of age were systematically evaluated, but they did not provide incremental prognostic value once tumor volume and metastatic status were included. This likely reflects collinearity with tumor burden and dissemination, demonstrating the strength of a data-driven feature selection process. Germline/genetic information (eg, *TP53*) could not be included due to inconsistent availability and legal constraints but remains a promising avenue for future models.

Subgroup analysis by COG stage highlighted the model's clinical relevance. Within advanced stages, ML-derived high-risk groups (based on median predicted log-risk) had significantly inferior survival (stage 3 HR 12.0, *P* = .0027; stage 4 HR 4.6, *P* = .0155), whereas no separation was seen in early stages (COG 1-2), likely due to very low event rates and ceiling effects. Notably, in COG stage 2, both events occurred in patients identified as high risk by the model. In COG stage 3, the model differentiated patients who may benefit from intensified adjuvant therapy or closer surveillance vs those at lower risk who may be eligible for avoiding overtreatment [3].

Absolute survival probabilities were derived via the Breslow estimator [30, 31]. While this assumes proportional hazards and should be interpreted with caution, the model itself is not constrained by this assumption. Ranking-based metrics (C-index) and SHAP explanations remain unaffected, preserving the reliability of relative risk predictions.

Direct benchmarking against existing systems such as pS-GRAS or COG for calibration or decision-curve analysis was not feasible, as these systems are categorical. Instead, the model complements existing frameworks by providing continuous individualized predictions. Calibtration was further assessed via time-dependent Brier scores (IBS 0.09) and visual plots at 1 and 3 years (Figs. S8 and S9 [27]), which showed reasonable agreement despite a small test-set size.

Several limitations merit acknowledgment. First, sample size was constrained by the rarity of pACT, limiting subgroup analyses and event counts in early stages. Second, external validation remains essential to assess generalizability across different clinical and geographic settings, including high-incidence settings; efforts toward multinational validation are ongoing through the European EXPeRT group [6] and the EU-funded Partner4VRT project. Third, only complete cases were analyzed, as imputation of clinically critical variables (tumor volume, resection, staging) was considered unreliable; while this may introduce selection bias, it preserves interpretability and clinical validity. Finally, ML approaches are not infallible. The model may still produce incorrect predictions in individual cases, particularly for patients whose feature profiles differ from those seen during training [32]. Individual predictions must therefore be interpreted with clinical judgment [33].

## Conclusions

This study demonstrates that interpretable, ML-based survival modeling can yield robust, clinically meaningful survival predictions in pACTs, particularly for patients with advanced nonmetastatic disease. By capturing nonlinear relationships and enabling individualized risk estimates, the model provides a nuanced complement to existing classification systems and may support more personalized decisions regarding adjuvant therapy and surveillance strategies.

## Data Availability

Restrictions apply to the availability of some or all data generated or analyzed during this study to preserve patient confidentiality or because they were used under license. The corresponding author will on request detail the restrictions and any conditions under which access to some data may be provided.

## Abbreviations

ACC: adrenocortical carcinoma
ACT: adrenocortical tumor
C-index: concordance index
CI: confidence interval
cM: clinical metastatic status
COG: Children's Oncology Group
HR: hazard ratio
IBS: integrated Brier score
ML: machine learning
pACT: pediatric adrenocortical tumor
pS-GRAS: pediatric S-GRAS
SHAP: SHapley Additive exPlanations
XGBoost: Extreme Gradient Boosting

## References

[1] Wasserman JD, Novokmet A, Eichler-Jonsson C, et al Prevalence and functional consequence of TP53 mutations in pediatric adrenocortical carcinoma: a children's oncology group study. J Clin Oncol. 2015;33(6):602‐609. 25584008 10.1200/JCO.2013.52.6863 PMC4517369

[2] Siegel DA, King J, Tai E, Buchanan N, Ajani UA, Li J. Cancer incidence rates and trends among children and adolescents in the United States, 2001-2009. Pediatrics. 2014;134(4):e945‐e955. 25201796 10.1542/peds.2013-3926 PMC4536809

[3] Kuhlen M, Mier P, Kunstreich M, et al Locally advanced adrenocortical carcinoma in children and adolescents—enigmatic and challenging cases. Cancers (Basel). 2023;15(17):4296. 37686571 10.3390/cancers15174296 PMC10486626

[4] Rodriguez-Galindo C, Figueiredo BC, Zambetti GP, Ribeiro RC. Biology, clinical characteristics, and management of adrenocortical tumors in children. Pediatr Blood Cancer. 2005;45(3):265‐273. 15747338 10.1002/pbc.20318

[5] Rodriguez-Galindo C, Krailo MD, Pinto EM, et al Treatment of pediatric adrenocortical carcinoma with surgery, retroperitoneal lymph node dissection, and chemotherapy: the Children's oncology group ARAR0332 protocol. J Clin Oncol. 2021;39(22):2463‐2473. 33822640 10.1200/JCO.20.02871 PMC8462560

[6] Cecchetto G, Ganarin A, Bien E, et al Outcome and prognostic factors in high-risk childhood adrenocortical carcinomas: a report from the European cooperative study group on pediatric rare tumors (EXPeRT). Pediatr Blood Cancer. 2017;64(6):e26368. 10.1002/pbc.26368 27957799

[7] Picard C, Faure-Conter C, Leblond P, et al Exploring heterogeneity of adrenal cortical tumors in children: the French pediatric rare tumor group (Fracture) experience. Pediatr Blood Cancer. 2020;67(2):e28086. 31738008 10.1002/pbc.28086

[8] Dall'Igna P, Virgone C, De Salvo GL, et al Adrenocortical tumors in Italian children: analysis of clinical characteristics and P53 status. Data from the national registries. J Pediatr Surg. 2014;49(9):1367‐1371. 25148739 10.1016/j.jpedsurg.2014.03.006

[9] Kuhlen M, Kunstreich M, Wudy SA, et al Outcome for pediatric adreno-cortical tumors is best predicted by the COG stage and five-item microscopic score-report from the German MET studies. Cancers (Basel). 2022;15(1):225. 36612221 10.3390/cancers15010225 PMC9818514

[10] Virgone C, Roganovic J, Vorwerk P, et al Adrenocortical tumours in children and adolescents: the EXPeRT/PARTNER diagnostic and therapeutic recommendations. Pediatr Blood Cancer. 2021;68(S4):e29025. 34174161 10.1002/pbc.29025

[11] Wieneke JA, Thompson LD, Heffess CS. Adrenal cortical neoplasms in the pediatric population: a clinicopathologic and immunophenotypic analysis of 83 patients. Am J Surg Pathol. 2003;27(7):867‐881. 12826878 10.1097/00000478-200307000-00001

[12] Picard C, Orbach D, Carton M, et al Revisiting the role of the pathological grading in pediatric adrenal cortical tumors: results from a national cohort study with pathological review. Mod Pathol. 2019;32(4):546‐559. 30401946 10.1038/s41379-018-0174-8

[13] Lopez-Nunez O, Virgone C, Kletskaya IS, et al Diagnostic utility of a modified reticulin algorithm in pediatric adrenocortical neoplasms. Am J Surg Pathol. 2024;48(3):309‐316. 38155550 10.1097/PAS.0000000000002174 PMC10876174

[14] Riedmeier M, Agarwal S, Antonini S, et al Assessment of prognostic factors in pediatric adrenocortical tumors: the modified pediatric S-GRAS score in an international multicenter cohort-a work from the ENSAT-PACT working group. Eur J Endocrinol. 2024;191(1):64‐74. 38924056 10.1093/ejendo/lvae079

[15] Yang Y, Wang X, Wu L, Zhao S, Chen R, Yu G. Identification and validation of susceptibility modules and hub genes of adrenocortical carcinoma through WGCNA and machine learning. Discov Oncol. 2025;16(1):663. 40317315 10.1007/s12672-025-02396-4 PMC12049343

[16] Marquardt A, Landwehr LS, Ronchi CL, et al Identifying new potential biomarkers in adrenocortical tumors based on mRNA expression data using machine learning. Cancers (Basel). 2021;13(18):4671. 34572898 10.3390/cancers13184671 PMC8469239

[17] Yan X, Guo ZX, Yu DH, et al Identification and validation of a novel prognosis prediction model in adrenocortical carcinoma by integrative bioinformatics analysis, statistics, and machine learning. Front Cell Dev Biol. 2021;9:671359. 34164395 10.3389/fcell.2021.671359 PMC8215582

[18] Martin-Hernandez R, Espeso-Gil S, Domingo C, et al Machine learning combining multi-omics data and network algorithms identifies adrenocortical carcinoma prognostic biomarkers. Front Mol Biosci. 2023;10:1258902. 38028548 10.3389/fmolb.2023.1258902 PMC10658191

[19] Tian X, Xu WH, Anwaier A, et al Construction of a robust prognostic model for adult adrenocortical carcinoma: results from bioinformatics and real-world data. J Cell Mol Med. 2021;25(8):3898‐3911. 33626208 10.1111/jcmm.16323 PMC8051734

[20] Saygili ES, Elhassan YS, Prete A, Lippert J, Altieri B, Ronchi CL. Machine learning-based survival prediction tool for adrenocortical carcinoma. J Clin Endocrinol Metab. 2025;110(10):e3185‐e3192. 39950976 10.1210/clinem/dgaf096 PMC12448644

[21] Tang J, Fang Y, Xu Z. Establishment of prognostic models of adrenocortical carcinoma using machine learning and big data. Front Surg. 2022;9:966307. 36684185 10.3389/fsurg.2022.966307 PMC9857757

[22] Roganovic J, Virgone C, Ben-Ami T, et al Paediatric very rare tumours registration and management in European countries with low health expenditure average rates. Clin Transl Oncol. 2025;27(4):1779‐1788. 39225960 10.1007/s12094-024-03674-3 PMC12000181

[23] Zhou L, Zhu Q, Chen Q, Wang P, Huang H. Predicting hospital outpatient volume using XGBoost: a machine learning approach. Sci Rep. 2025;15(1):17028. 40379678 10.1038/s41598-025-01265-y PMC12084583

[24] Dong T, Oronti IB, Sinha S, et al Enhancing cardiovascular risk prediction: development of an advanced Xgboost model with hospital-level random effects. Bioengineering (Basel). 2024;11(10):1039. 39451414 10.3390/bioengineering11101039 PMC11505330

[25] Chen T, Guestrin C. XGBoost: a scalable tree boosting system. presented at: Proceedings of the 22nd ACM SIGKDD International Conference on Knowledge Discovery and Data Mining. San Francisco, CA: Association for Computing Machinery, 2016.

[26] Lundberg SM, Erion G, Chen H, et al From local explanations to global understanding with explainable AI for trees. Nat Mach Intell. 2020;2(1):56‐67. 32607472 10.1038/s42256-019-0138-9 PMC7326367

[27] Redlich A, Pfaehler E, Kunstreich M, et al Data from: Interpretable Machine Learning Model for Survival Prediction in Pediatric Adrenocortical Tumors. 10.5281/zenodo.17314034. Deposited 9 October 2025. PMC12838521 41608202

[28] Riedmeier M, Decarolis B, Haubitz I, et al Assessment of prognostic factors in pediatric adrenocortical tumors: a systematic review and evaluation of a modified S-GRAS score. Eur J Endocrinol. 2022;187(6):751‐763. 36193775 10.1530/EJE-22-0173

[29] Zambaiti E, Duci M, De Corti F, et al Clinical prognostic factors in pediatric adrenocortical tumors: a meta-analysis. Pediatr Blood Cancer. 2021;68(3):e28836. 33306282 10.1002/pbc.28836

[30] Lin DY . On the breslow estimator. Lifetime Data Anal. 2007;13(4):471‐480. 17768681 10.1007/s10985-007-9048-y

[31] Moncada-Torres A, van Maaren MC, Hendriks MP, Siesling S, Geleijnse G. Explainable machine learning can outperform cox regression predictions and provide insights in breast cancer survival. Sci Rep. 2021;11(1):6968. 33772109 10.1038/s41598-021-86327-7 PMC7998037

[32] Lu SC, Swisher CL, Chung C, Jaffray D, Sidey-Gibbons C. On the importance of interpretable machine learning predictions to inform clinical decision making in oncology. Front Oncol. 2023;13:1129380. 36925929 10.3389/fonc.2023.1129380 PMC10013157

[33] Nohara Y, Matsumoto K, Soejima H, Nakashima N. Explanation of machine learning models using shapley additive explanation and application for real data in hospital. Comput Methods Programs Biomed. 2022;214:106584. 34942412 10.1016/j.cmpb.2021.106584

